# The Potential for Healthy Checkout Policies to Advance Nutrition Equity

**DOI:** 10.3390/nu13114181

**Published:** 2021-11-22

**Authors:** Jennifer Falbe, Justin S. White, Desiree M. Sigala, Anna H. Grummon, Sarah E. Solar, Lisa M. Powell

**Affiliations:** 1 Human Development Program, Department of Human Ecology, University of California, Davis, CA 95616, USA; sesolar@ucdavis.edu; 2 Philip R. Lee Institute for Health Policy Studies, University of California, San Francisco, CA 94158, USA; Justin.White@ucsf.edu; 3 Department of Molecular Biosciences, School of Veterinary Medicine, University of California, Davis, CA 95616, USA; dmsigala@ucdavis.edu; 4 Department of Nutrition, Harvard TH Chan School of Public Health, Boston, MA 02115, USA; agrummon@hsph.harvard.edu; 5 Division of Health Policy and Administration, School of Public Health, University of Illinois Chicago, Chicago, IL 60305, USA; powelll@uic.edu

**Keywords:** checkout, policy, product placement, obesity, nutrition, retail, marketing, disparities, race, income

## Abstract

Background: As the only place in a store where all customers must pass through and wait, the checkout lane may be particularly influential over consumer purchases. Because most foods and beverages sold at checkout are unhealthy (e.g., candy, sweets, sugar-sweetened beverages, and salty snacks), policymakers and advocates have expressed growing interest in healthy checkout policies. To understand the extent to which such policies could improve nutrition equity, we assessed the prevalence and sociodemographic correlates of purchasing items found at (i.e., from) checkout. Methods: We assessed self-reported checkout purchasing and sociodemographic characteristics in a national convenience sample of adults (*n* = 10,348) completing an online survey in 2021. Results: Over one third (36%) of participants reported purchasing foods or drinks from checkout during their last grocery shopping trip. Purchasing items from checkout was more common among men; adults < 55 years of age; low-income consumers; Hispanic, non-Hispanic American Indian or Alaska Native, and non-Hispanic Black consumers; those with a graduate or professional degree; parents; and consumers diagnosed with type 2 diabetes or pre-diabetes (*p*-values < 0.05). Conclusions: Purchasing foods or beverages from store checkouts is common and more prevalent among low-income and Hispanic, American Indian or Alaska Native, and Black consumers. These results suggest that healthy checkout policies have the potential to improve nutrition equity.

## 1. Introduction

Two thirds of calories in the US diet come from grocery stores [1,2], making the retail food environment a key opportunity for improving diet quality. Multiple reviews have found that the characteristics of store food environments influence consumer purchases, especially product placement and pricing [3,4,5]. The store food environment may also affect health outcomes. For example, higher exposure to store displays for sugar-sweetened beverages and foods high in added sugars has been associated with higher customer body mass index [6]. 

Checkout lanes represent a particularly promising target for intervention. Checkout lanes are the only place in the store where all customers must pass through and wait. Moreover, checkout lanes are known for high levels of impulse purchases [7,8]. Thus, processed food manufacturers pay stores large sums of money to place their products at checkout [9]. Indeed, the most common foods and beverages found at checkout include candy, sugar-sweetened beverages, salty snacks, and sweets [10,11,12,13]. The food environment in checkout lanes also might contribute to prevailing inequities in dietary and health outcomes, such as higher consumption of unhealthy foods and beverages and higher prevalence of obesity in low-income, Black, and Hispanic populations [14,15,16,17,18,19]. One national study, for example, found that stores in low-income communities were less likely to carry fresh fruits and vegetables at checkout than stores in higher income communities [10]. 

Some stores in the US have made voluntary changes to improve the healthfulness of their checkouts. These changes have typically been short-term, narrow in scope (e.g., converting only one checkout lane per store), and not widely adopted. To address this issue, in 2021, Berkeley, California became the first jurisdiction in the US—and, to our knowledge, the first in the world—to implement a healthy checkout policy [20,21]. The ordinance prohibits sweetened beverages and foods containing >5 g of added sugars or >200 mg of sodium per serving from being displayed at checkout. The UK recently followed suit, adopting a healthy checkout policy that will be implemented in 2022 [22,23]. These policies have the potential to encourage healthier purchases. Assessments of voluntary standards in the UK restricting junk food at checkouts found that the standards were associated with fewer purchases of small packages of sweets and salty snacks commonly sold at checkout [24]. 

The extent to which healthy checkout policies improve consumer purchases, and ultimately dietary intake and chronic disease risk, depends in part on how frequently consumers purchase foods and beverages found in the checkout area (i.e., “from checkout”). Further, the extent to which checkout policies may advance nutrition equity depends upon which segments of the population are more likely to make food and beverage purchases from checkout. To date, limited published research has examined checkout behaviors. Therefore, the objective of this study was to assess the prevalence and frequency of purchasing from checkout and to identify sociodemographic correlates of this practice.

## 2. Materials and Methods

### 2.1. Participants Design

This study draws data from a national online sample of 15,502 US adults recruited in May–June 2021 to match the 2018 American Community Survey (ACS) 5-year estimates [25] for age (18–34, 35–54, ≥55 years), gender, race/ethnicity (Hispanic [any race], non-Hispanic (NH) White, NH Black, and NH Asian), and education (up to a high school diploma or equivalent, some college, at least a bachelor’s degree). The sample was recruited by Dynata, which maintains panels of US adults recruited using open enrollment and by-invitation-only methods [26]. 

### 2.2. Procedures

Participants provided informed consent and completed a screener to assess eligibility. Eligible participants were English-speaking US residents aged 18–99 years who reported purchasing items from restaurants ≥1 time per month prior to the pandemic and passed a Captcha. Participants then completed a 10–15 min Qualtrics survey. The primary purpose of the survey was to test the effect of warning labels for restaurant menu items high in added sugars in a hypothetical menu ordering task (manuscript under review). After completing this ordering task, participants answered questions about purchasing items from grocery store checkouts, as described below. This study was approved by the UC Davis Institutional Review Board. 

### 2.3. Measures

Two novel questions assessed purchasing from checkout: (1) “The last time you went to the grocery store, did you purchase any foods or drinks that you found in the checkout area? (yes or no)” and (2) “Before the pandemic, how often did you purchase something you found in the checkout area of a store? (3 or more times per week, 2 times per week, 1 time per week, 2–3 times per month, 1 time per month, or less than 1 time per month)”. 

The questionnaire assessed the following sociodemographic characteristics: gender, continuous age, race and ethnicity (Hispanic, American Indian or Alaska Native, Asian, Black, Native Hawaiian or other Pacific Islander, Middle Eastern or North African, or White), educational attainment (less than a high school diploma, high school diploma or GED, some college or associate’s degree, bachelor’s degree, or graduate or professional degree), total household income in the last 12 months before taxes (<$20,001, $20,001 to $150,000 in $15,000 increments, and >$150,000), being a parent or caregiver of a child age 0–17 years, and having been diagnosed with pre-diabetes or type 2 diabetes. An attention check question asked participants to select the current month. If a participant selected the incorrect month, they were classified as having failed the attention check question. 

For the analysis, age was categorized into 18–34, 35–54, and 55+ years. Race and ethnicity were grouped as Hispanic any race and NH American Indian or Alaska Native, NH Asian, NH Black, NH Multiracial, NH Native Hawaiian or other Pacific Islander, and NH White. Income was categorized into approximate quartiles based on the income categories, and diagnosis with either pre-diabetes or type 2 diabetes was combined into a single dichotomous variable. 

### 2.4. Analytic Sample

Because items assessing checkout behaviors were added after the survey’s launch, a total of 10,774 participants took a version of the questionnaire with checkout questions. Of the 10,774 participants, we excluded 381 for failing the attention check question and 45 for completing the survey in less than 30% of the median completion time, leaving an analytic sample of 10,348.

### 2.5. Statistical Analysis

Counts and percentages were used to present descriptive statistics regarding sociodemographic characteristics and checkout purchasing behaviors. A Spearman correlation coefficient was calculated to examine the extent to which educational attainment and income were correlated. Prior to conducting main analyses, we assessed whether random exposure to the restaurant warning label in the menu ordering task affected participant responses to the checkout questions using a Poisson regression model with a robust error variance [27] for the question assessing probability of purchasing from checkout during the last shopping trip and a chi-square test for the question assessing categorical frequency of checkout purchases. Results revealed no significant association between experimental condition in the menu ordering task and checkout purchasing behaviors (*p*-values > 0.05).

To examine the association between sociodemographic characteristics and probability of purchasing foods and drinks from checkout during the last shopping trip, we calculated prevalence ratios using Poisson regression models with a robust error variance [27]. The first model examined unadjusted bivariate associations between each characteristic and purchasing foods or drinks from checkout. The second model (i.e., the adjusted model) included all sociodemographic variables in the same model. Additionally, we used post hoc pairwise comparisons to examine differences in probability of purchasing foods and drinks from checkout between each category of education and between each category of income, using the Holm–Bonferroni procedure [28] to correct for multiple comparisons. Analyses used complete case analysis and were conducted using Stata/MP v15.1 in 2021 (StataCorp LLC, College Station, TX, USA).

## 3. Results

### 3.1. Sample Characteristics

The sample was 56% women, 19% Hispanic of any race, 1% NH American Indian or Alaska Native, 6% NH Asian, 16% NH Black, 1% Multiracial, <1% NH Native Hawaiian or other Pacific Islander, and 57% NH White (Table 1). Nearly 40% had no more than a high school degree or GED, 38% had some college or associate’s degree, and 22% had at least a bachelor’s degree. There was a moderate correlation between participant educational attainment and household income (Spearman rho = 0.41; *p*-value < 0.001).

### 3.2. Purchasing from Checkout

More than one third (36%) of participants reported purchasing foods or drinks found in the checkout area during their last grocery store trip (Table 2). Additionally, 51% reported that, before the pandemic, they purchased something found at checkout at least once per month, with 29% reporting doing so at least once per week (Table 2). 

### 3.3. Sociodemographic Characteristics Associated with Purchasing Foods or Drinks from Checkout

Table 3 presents unadjusted and adjusted associations between sociodemographic characteristics and purchasing foods or drinks from checkout during the last grocery shopping trip. In unadjusted and adjusted models, men were more likely than women, and younger adults (age 18–54) were more likely than older adults (age ≥ 55), to report purchasing foods or drinks from checkout. Adjusted estimates for race and ethnicity were substantially attenuated compared to estimates in the unadjusted models, but the adjusted probability of purchasing foods or drinks from checkout remained significantly higher than NH White participants for Hispanic (1.1 times higher), NH American Indian or Alaska Native (1.4 times higher), and NH Black participants (1.3 times higher). In the unadjusted and adjusted models, being a parent and having had a diagnosis of type 2 diabetes or pre-diabetes were associated with a higher probability of purchasing foods or drinks from checkout. In the unadjusted models, there was a U-shaped association of both educational attainment and income with purchasing foods or drinks from checkout, wherein those with the lowest and highest levels were the most likely to purchase from checkout. In the adjusted models, however, participants with educational attainment levels of less than a high school diploma, a high school diploma or GED, and some college were less likely to purchase foods or drinks from checkout than those with a graduate or professional degree. There were no significant differences in the likelihood of purchasing from checkout between the lower three levels of educational attainment (less than a high school diploma, a high school diploma or GED, and some college or associate’s degree). Nor was there a significant difference between either of the two lowest levels of educational attainment and a bachelor’s degree. Additionally, in the adjusted models, the U-shaped relationship between income and purchasing from checkout was no longer present; instead, the lower the income level, the more likely participants were to purchase foods or drinks from checkout.

## 4. Discussion

In this large, national sample of US adults, more than one in three (36%) participants reported purchasing foods or drinks from checkout during their last grocery shopping trip. Further, most participants (51%) reported purchasing something from checkout at least monthly prior to the pandemic. Consistent with the hypothesis that stocking checkout with poor-quality foods and beverages may contribute to inequities, we found that the lowest-income participants were more likely to purchase foods and drinks from checkout than the highest-income participants, as were Hispanic, NH American Indian or Alaska Native, and NH Black participants compared to NH White participants. Further, men, adults under age 55, parents, and those reporting a diagnosis of type 2 diabetes or pre-diabetes were more likely to report purchasing foods or drinks from checkout. Those with less than a bachelor’s degree were less likely to purchase foods or drinks from checkout than those with a graduate or professional degree. This is the first study, to our knowledge, to use a national US sample to examine the prevalence and frequency of purchasing items from checkout and to identify groups among which checkout purchases were more common. This information is key for understanding the extent to which healthy checkout policies can improve diet quality and reduce nutrition and health inequities.

We are unaware of any other studies in the US that examine the reported purchasing of foods or beverages from checkout. However, one study in which researchers observed behaviors during specific times at three South Bronx supermarkets in New York City found that only 4% of customers purchased an item from checkout [29]. The authors noted, though, that the checkouts in these stores were compact, moved quickly, and were not representative of checkouts more broadly. In contrast to our results, a study in the UK that analyzed household purchases from nine leading supermarkets found no association between socioeconomic status and number of purchases of foods commonly sold at checkout [30]. However, the mentioned study was unable to determine whether foods commonly sold at checkout were actually obtained at checkout or elsewhere in the store. 

An unexpected finding from our study was that, in the adjusted models, those with the highest education level (graduate or professional degree) were more likely to purchase foods or drinks from checkout than those with less than a bachelor’s degree. This observation was unexpected because the relationship between education and health behaviors is typically similar to that between income and health behaviors. It is possible that those with the highest education levels lived near, and consequently shopped at, different types of stores that stocked healthier foods and beverages at checkout. We did not, however, observe any differences in purchasing from checkout between the following educational attainment groups: less than a high school diploma, high school diploma or GED, and some college or associate’s degree. 

In our sample, a sizable proportion of the population reported purchasing foods or beverages from checkout, and this practice was more common among low-income and Hispanic, American Indian or Alaska Native, and Black consumers. These results suggest that healthy checkout policies have the potential to improve diet quality and promote nutrition equity. Although there are no evaluations of mandatory checkout policies such as the ones recently adopted in Berkeley and the UK, studies of voluntary initiatives indicate that healthy checkout policies may improve the nutritional quality of store purchases. The strongest evidence comes from a natural experiment in the UK, where multiple chains implemented voluntary checkout standards. Using data from household purchases and comparing UK stores with checkout standards to those without, researchers found that checkout standards reduced sales of unhealthy checkout foods by 17% [24]. Other evaluations of voluntary checkout initiatives have found that the initiatives increased purchases of healthier products or decreased purchases of unhealthy products [29,31,32,33,34,35,36]. 

A strength of this study is the large and diverse national sample of participants. Limitations include the fact that purchasing behaviors were self-reported; data were cross-sectional; and the questionnaire did not assess types of foods or beverages purchased from checkout. Additionally, we did not recruit a probability sample, so the results may not generalize to the US as a whole, although the sample was recruited to approximately match the US adult population with respect to categories of age, gender, race/ethnicity, and educational attainment. 

## 5. Conclusions

Purchasing foods or drinks from checkout was common in this national sample of US adults. Additional research is needed to understand the frequency with which specific types of foods and beverages are purchased from checkout, checkout purchases are made from different store types (e.g., supermarket, drugstore), and checkout foods and beverages are purchased by or for children. This study’s finding that purchasing foods or drinks from checkout was more prevalent among low-income and Hispanic, NH American Indian or Alaska Native, and NH Black consumers, together with prior research documenting the poor quality of foods and beverages at checkout, indicate that healthy checkout policies hold promise for improving nutrition and health equity. Additionally, this study’s findings that parents and those diagnosed with diabetes or pre-diabetes were more likely to purchase foods or drinks from checkout suggest that healthy checkout policies may have broad reach to adults, children, and those most at risk for nutrition-related health harms. To meaningfully promote health and equity, healthy checkout policies will likely need to be comprehensive and apply to a wide variety of store types—including store types from which low-income and racially and ethnically diverse households purchase the majority of their calories. Such policies should be rigorously evaluated to determine their impacts on consumer behavior and nutrition equity. 

## Figures and Tables

**Table 1:** Sociodemographic characteristics of a national sample of US adults.

Characteristic	*n* (%)
*N*	10,348
Gender	
Man	4528 (44%)
Non-binary or gender nonconforming	46 (<1%)
Woman	5774 (56%)
Age	
18–34 years	2644 (26%)
35–54 years	3053 (30%)
55+ years	4651 (45%)
Race/ethnicity	
Hispanic, any race	2012 (19%)
NH American Indian or Alaska Native	56 (1%)
NH Asian	667 (6%)
NH Black	1605 (16%)
NH Multiracial	94 (1%)
NH Native Hawaiian or other Pacific Islander	13 (<1%)
NH White	5901 (57%)
Education level	
Less than high school or GED	339 (3%)
High school or GED	3781 (37%)
Some college or associate’s degree	3950 (38%)
Bachelor’s degree	1396 (13%)
Graduate or professional degree	882 (9%)
Annual household income before taxes	
≤$35,000	3163 (31%)
$35,001–65,000	2911 (28%)
$65,001–95,000	1838 (18%)
>$95,000	2385 (23%)
Parent or caregiver of child <18 years	2680 (26%)
Diagnosed with pre-diabetes or type 2 diabetes	2233 (22%)
Region	
West	2226 (22%)
Midwest	1979 (19%)
Northeast	2037 (20%)
South	4087 (40%)
US Territory	13 (<1%)

Note: Missing responses were not included in the denominator for calculating percentages. Data were missing for only income, parent or caregiver status, diabetes diagnosis, and region and ranged from 0.1 to 3% of observations. GED—general education development; NH—non-Hispanic.

**Table 2:** Proportion of participants purchasing items from checkout and frequency of this practice in a national sample of US adults.

Questionnaire Item	*n* (%)
“The last time you went to the grocery store, did you purchase any foods or drinks that you found in the checkout area?” (*n* = 10,343)	
Yes	3688 (36%)
No	6655 (64%)
“Before the pandemic, how often did you purchase something you found in the checkout area of a store?” (*n* = 10,337)	
Less than 1 time per month	5037 (49%)
1 time per month	1035 (10%)
2–3 times per month	1251 (12%)
1 time per week	1242 (12%)
2 times per week	1000 (10%)
3 or more times per week	772 (7%)

**Table 3:** Prevalence and prevalence ratios of purchasing foods or drinks found in the checkout area (i.e., from checkout) during the last grocery shopping trip by sociodemographic characteristics.

	Prevalence of Purchasing from Checkout	Prevalence Ratio (95% CI) ^1^ of Purchasing from Checkout	
	*n* (%)	Unadjusted Bivariate (*n* ≥ 10,277) ^2^	Adjusted ^3^ (*n* = 10,214)
Gender			
Man	1817 (40%)	1.25 (1.19, 1.32) ***	1.12 (1.07, 1.18) ***
Gender nonconforming	17 (37%)	1.15 (0.79, 1.68)	0.76 (0.51, 1.14)
Woman	1854 (32%)	ref	ref
Age			
18–34	1700 (64%)	5.76 (5.28, 6.27) ***	4.48 (4.05, 4.94) ***
35–54	1468 (48%)	4.30 (3.93, 4.70) ***	3.27 (2.96, 3.61) ***
55+	520 (11%)	ref	ref
Race and ethnicity			
Hispanic, any race	1015 (51%)	2.10 (1.97, 2.23) ***	1.12 (1.05, 1.19) ***
NH American Indian or Alaska Native	42 (75%)	3.11 (2.66, 3.65) ***	1.37 (1.19, 1.59) ***
NH Asian	212 (32%)	1.32 (1.17, 1.49) ***	0.90 (0.81, 1.01) ^†^
NH Black	946 (59%)	2.45 (2.30, 2.60) ***	1.25 (1.18, 1.33) ***
NH Multiracial	45 (48%)	1.99 (1.60, 2.47) ***	1.20 (0.98, 1.47) ^†^
NH Native Hawaiian or other Pacific Islander	7 (54%)	2.24 (1.35, 3.71) **	1.09 (0.64, 1.86)
NH White	1421 (24%)	ref	ref
Educational attainment			
Less than a high school diploma	164 (49%)	1.10 (0.96, 1.25) ^a^	0.85 (0.74, 0.97) *
High school diploma or GED	1305 (35%)	0.78 (0.72, 0.85) *** ^b^	0.90 (0.83, 0.98) *
Some college or associate’s degree	1186 (30%)	0.68 (0.62, 0.74) *** ^c^	0.86 (0.80, 0.94) *** ^a^
Bachelor’s degree	643 (46%)	1.04 (0.95, 1.14) ^a^	0.98 (0.91, 1.07) ^b^
Graduate or professional degree	390 (44%)	ref	ref
Annual household income before taxes			
≤$35,000	1310 (41%)	1.10 (1.03, 1.17) ** ^a^	1.24 (1.16, 1.33) *** ^a^
$35,001–$65,000	885 (30%)	0.80 (0.75, 0.87) *** ^b^	1.07 (1.00, 1.15) ^† b^
$65,001–$95,000	580 (32%)	0.83 (0.77, 0.91) *** ^b^	1.06 (0.98, 1.14) ^b^
>$95,000	902 (38%)	ref	ref
Parent or caregiver of child <18 years of age			
Parent of a child	1710 (64%)	2.48 (2.36, 2.60) ***	1.50 (1.43, 1.57) ***
Not a parent of a child	1958 (26%)	ref	ref
Type 2 diabetes or pre-diabetes diagnosis			
Diagnosis	842 (38%)	1.08 (1.01, 1.14) *	1.26 (1.19, 1.33) ***
No diagnosis	2820 (35%)	ref	ref

Note: The outcome was assessed with the question, “The last time you went to the grocery store, did you purchase any foods or drinks that you found in the checkout area?” (yes/no). ^1^ Prevalence ratios, 95% CI, and *p*-values were calculated from Poisson regression models with robust standard errors. ^2^ The sample size for models in which independent variables were gender, age, race/ethnicity, and education was 10,343. Sample sizes for models in which the independent variables were income, parent/caregiver, and diagnosis with type 2 diabetes/pre-diabetes were 10,292, 10,282, and 10,277, respectively. ^3^ The adjusted model included all characteristics in Table 3 as independent variables. *** *p* < 0.001, ** *p* < 0.01, * *p* < 0.05, ^†^ *p* < 0.10. ^a^ differs significantly from ^b^ and ^c^, and ^b^ differs significantly from ^c^ in post hoc pairwise comparisons using the Holm–Bonferroni procedure to correct for multiple comparisons. CI—confidence interval; NH—non-Hispanic.

## Data Availability

The data presented in this study are available to researchers for non-commercial use upon reasonable request from the corresponding author. The data are not publicly available to protect participant privacy.
